# Preparing at the Local Level for Events Involving Weapons of Mass Destruction

**DOI:** 10.3201/eid0809.010520

**Published:** 2002-09

**Authors:** Marna L. Hoard, Janet M. Williams, James C. Helmkamp, Paul M. Furbee, William G. Manley, Floyd K. Russell

**Affiliations:** West Virginia University, Morgantown, West Virginia

**To the Editor:** The use of hijacked airplanes in the attacks on the World Trade Center and the Pentagon on September 11, 2001, clearly illustrated the immediate and massive destruction that can result from a well-orchestrated, long-planned, and purposeful terrorist act. Weapons of mass destruction (WMD) events (i.e., biological, nuclear, or chemical attacks) present different challenges than other incidents involving mass casualties (e.g., chemical spills, transportation mishaps, or natural disasters). Persons involved in a biological weapons attack, for example, may take days to develop symptoms and seek medical care (1); a large geographic area may be affected, or persons may travel long distances and unwittingly infect others, including hospital personnel (2). Furthermore, traditional hazardous materials and emergency medical procedures may be inadequate to respond to a WMD event (3–5). As events of September 11 and its aftermath make clear, medical public health systems were not optimally prepared. An effective response to a WMD event focuses on two key areas: joint efforts between the medical community and public health agencies and better trained and coordinated first responders (i.e., law enforcement, public safety, hospital personnel, and public health officials (1–3).

In early 2001, telephone interviews with West Virginia county health directors (CHDs) or their equivalent were conducted to ascertain the level of collaboration between their departments and local hospitals in regard to WMD preparedness and a coordinated medical and public health response. Forty-four (90%) of 49 CHDs completed the interview. One of the 49 responding CHDs is responsible for a six-county area, thus accounting for the state’s 55 counties.

Fewer than half (20 of 44) of the respondents have provided contact information to local hospitals, and barely 20% have reciprocal information. Twenty-one percent were either unaware of a policy for WMD preparedness or reported that it was being handled by another agency. Although 72% of CHDs had attended WMD training, only 14% of the training was in conjunction with hospitals. While nearly two thirds rated their communication with hospitals as moderate to strong, a similar proportion stated they had no protocol for communicating with hospitals about a WMD event. Eighty-nine percent of CHDs reported that no new collaborative efforts were directed towards the early identification of new or emerging infectious diseases possibly related to bioterrorism. However, approximately one third of the CHDs thought they should take initiative in this matter. Over 60% indicated that primary responsibility for identifying biological agents rested in another agency or was not the sole responsibility of the CHD. Further, 20% indicated they were weak or untrained in this area and thought that development and implementation of policies, procedures, and training were needed. While 93% of CHDs felt joint training with hospitals would be beneficial, particularly in defining their respective roles in a WMD scenario, many cited manpower and scheduling constraints for such joint training sessions. Overall, CHDs reported weak relationships with area hospitals, but thought that development or improvement of policies and procedures through regular meetings and training would help prepare and plan for a WMD event.

The results of this survey suggest that before September 11, West Virginia CHDs and local hospitals had little collaboration in preparing to respond to a WMD event. Despite the recent terrorist activities, local health departments and hospitals are still reluctant to spend resources in preparation for events with a low probability of occurring, such as WMD incidents. The local health departments and hospitals think that other pressing programs will be jeopardized (6–8). Many federal and state initiatives are under way to enhance the public health infrastructure and its preparation and response to bioterrorism. Improving on programs to meet daily operational challenges, as well as those presented by a WMD event, must include the expertise of local health departments and hospitals and encourage the creation of innovative, cost-effective preparedness programs at the local level (9,10). Future research should be conducted in areas of resource education and training, allocation and sharing, personnel, and policy. This research will indicate if existing programs should be improved and if new programs should be instituted.
